# Clinical Analysis of 17 Cases of Borderline Ovarian Tumors During Pregnancy

**DOI:** 10.3389/fonc.2022.934751

**Published:** 2022-07-13

**Authors:** Mingdan Wang, Yue Li, Tongtong Xu, Chen Shi, Lili Jiang, Kuiran Liu

**Affiliations:** Department of Obstetrics and Gynecology, Shengjing Hospital of China Medical University, Shenyang, China

**Keywords:** borderline ovarian tumor, pregnancy, diagnosis, treatment, prognosis

## Abstract

**Objective:**

The study retrospectively analyzed the clinical characteristics and prognosis of 17 cases of pregnancy complicated by borderline ovarian tumors to provide help for clinical workers.

**Materials and Methods:**

The clinicopathological data of 17 patients with ovarian borderline tumors during pregnancy at the Shengjing Hospital of China Medical University from January 2015 to June 2021 were collected and analyzed retrospectively. The average age of the patients was 31.82 years (25–45 years), the average number of pregnancies was 2.06 times (1–6 times), and the average number of births was 0.41 times (0–1 time).

**Results:**

Among the 17 patients, 4 were diagnosed in the first trimester, 2 in the second trimester, and 11 in the third trimester. Most of the first symptoms were cysts, cyst enlargement, or cyst rupture. Among them, 3 cases (1 in the first trimester and 2 in the second trimester) continued pregnancy after a conservative operation, 9 cases underwent cesarean section and a conservative operation simultaneously, and the mother and child had a good outcome. Two cases underwent conservative operations and induced abortion, and 1 case underwent an ectopic pregnancy operation at the same time. The prognosis of the patients was good without recurrence.

**Conclusion:**

Preoperative diagnosis of borderline ovarian tumors in pregnancy is delayed, and imaging and tumor markers are not specific. The coincidence rate between intraoperative frozen pathology and postoperative paraffin pathology was not high. Borderline tumors are mainly treated by surgery, and the prognosis for mothers and infants is good.

## 1 Introduction

An ovarian tumor is a common tumor of the female reproductive system. With the extension of the reproductive age of women and the popularization and deepening of perinatal health care, the incidence and detection rate of pregnancy with ovarian tumors are also gradually increasing (1). Most ovarian tumors during pregnancy are physiological cysts or benign tumors, and less malignant or borderline tumors. Compared with ovarian malignancies, patients with borderline ovarian tumors are diagnosed at a younger age, and about one-third of patients are diagnosed before the age of 40 (2). Although systematic ultrasonography is performed during pregnancy, the diagnosis and treatment of pregnancy complicated by ovarian borderline tumors are still unclear. This study retrospectively analyzed 17 cases of pregnancy complicated by borderline ovarian tumor admitted to the Shengjing Hospital of China Medical University from January 2015 to June 2021 and followed up for 1 to 7 years. By analyzing the clinicopathological characteristics and prognosis of pregnancy complicated with borderline ovarian tumors during pregnancy, we hope to provide help to clinicians.

## 2 Materials and methods

### 2.1 Clinical Data

Clinical data were collected from 17 pregnant patients with borderline ovarian tumors from January 2015 to June 2021 at the Shengjing Hospital of China Medical University; all cases were pathologically diagnosed as borderline tumors or focal borderline tumors. The average age of these patients was 31.82 years (range, 25 to 45 years) with a mean of 2.06 pregnancies (range, 1 to 6) and a mean of 0.41 deliveries (range, 0 to 1).

### 2.2 Methods

The information about the patient, including the age, reproductive history, confirmed gestational weeks, clinical symptoms, tumor markers, imaging characteristics, surgical methods, gestational weeks of termination of pregnancy, tumor pathological types, maternal and infant outcomes, and follow-up treatment, were collected in detail. The patients and their offspring were followed up, and then the clinical, imaging, and pathological characteristics of 17 patients were summarized, and the treatment effect and prognosis were analyzed. This aims to provide guidance and help for the clinical diagnosis and treatment of borderline ovarian tumors during pregnancy.

## 3 Results

### 3.1 Clinical Manifestations

**Figure 1:**
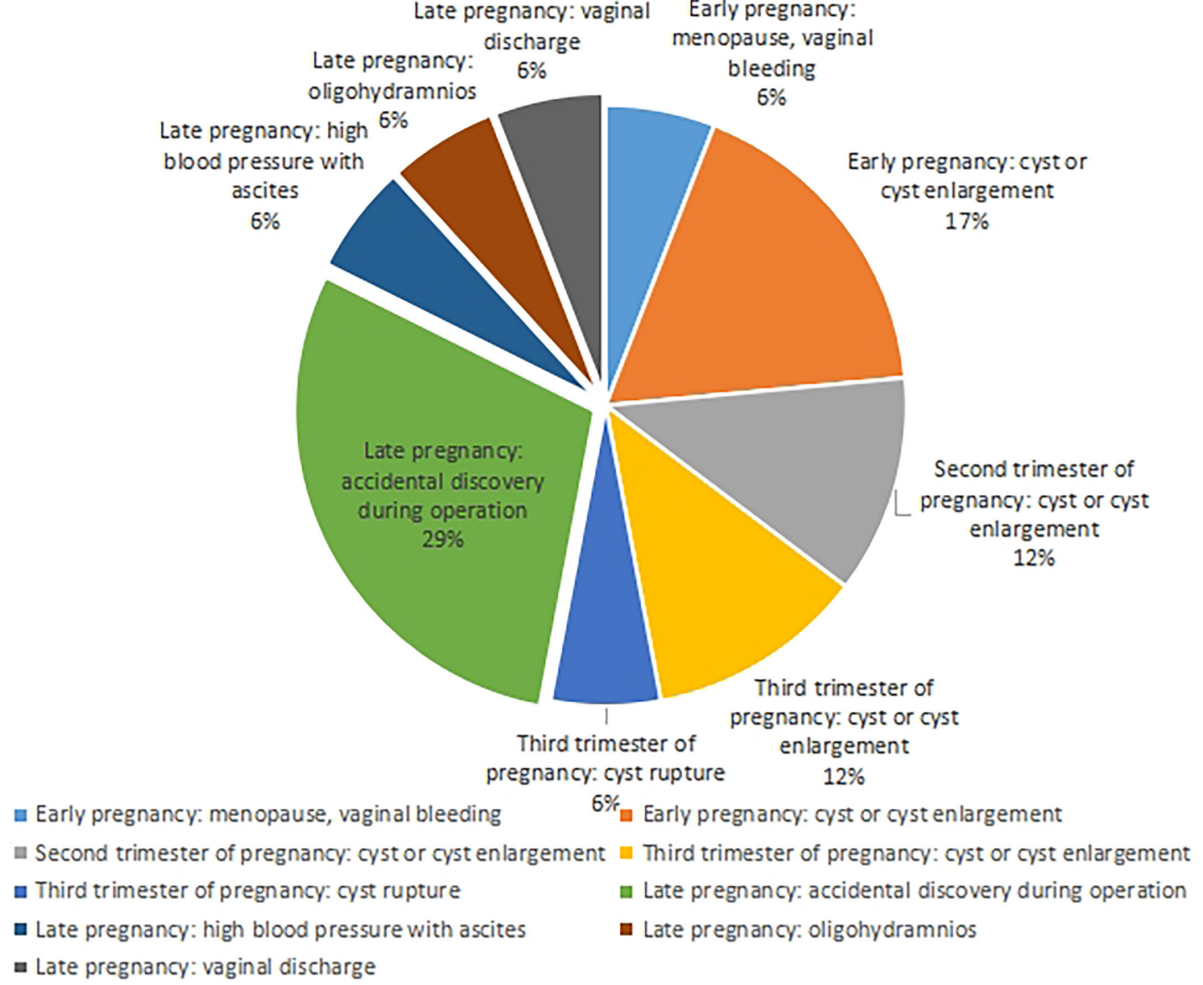
Clinical manifestations of 17 patients.

The main clinical manifestations of most patients were cysts, cyst enlargement, or cyst rupture (47%). Among the patients diagnosed in the first trimester, 3 cases (17%) were diagnosed because of an ovarian cyst, and 1 case (6%) complicated by ectopic pregnancy was diagnosed because of menopause and vaginal bleeding. Among the 2 cases (12%) diagnosed in the second trimester, they were all treated because of an ovarian cyst or cyst enlargement. Of the 11 patients in the third trimester, 2 cases (12%) had giant ovarian cysts or cyst enlargement, and 1 case (6%) had cyst rupture. Obstetric symptoms such as high blood pressure and ascites were found in 1 case (6%), less amniotic fluid in 1 case (6%), vaginal fluid in 1 case (6%), and premonitory delivery and elective cesarean section in 5 cases (29%) (**Figure 1**).

### 3.2 Diagnosis

#### 3.2.1 Imaging Diagnosis

Pelvic ultrasonography is the gold standard and first-line examination for the examination of adnexal masses during pregnancy (3). Among the 17 patients, 13 (76.5%) showed adnexal masses. The results of a pelvic MRI examination in some patients were consistent with those of the ultrasound examination. The patients with tumors found in the first trimester and no intention of pregnancy were examined by abdominal CT, and the CT examination showed no positive indication. Ultrasound is the main imaging diagnostic method during pregnancy. Among the 4 patients diagnosed in early pregnancy, 3 cases were treated because of the discovery of cysts or the enlargement of cysts. All the 3 patients showed that the diameter of the cysts was >10 cm Two cases showed cystic solids and 1 case had cystic. In another case, pelvic ultrasound was performed because of amenorrhea and vaginal bleeding, and a cystic mass with an appendix of 3.0 cm was accidentally found. In the second trimester of pregnancy, 2 patients were treated for finding cysts or cyst enlargement. Pelvic ultrasound showed cystic and solid masses with a diameter of <4 cm. Two of the 11 patients in the third trimester of pregnancy were diagnosed because of cysts or enlarged cysts. Ultrasound showed cystic masses with a diameter of >15 cm; 1 case was treated for cyst rupture, and ultrasound showed an 8.3 cm cystic mass. During pregnancy, ultrasound in 4 patients did not show an adnexal mass, which may be blocked in the bilateral adnexa due to uterine enlargement during pregnancy; the other 4 cases showed cystic or solid masses in the adnexal region in a routine ultrasound examination.

#### 3.2.2 Diagnosis of Tumor Markers

CA125 was the first choice of ovarian tumor marker. Among the 11 patients who detected serum CA125, 6 (54.5%) increased it (2 cases significantly increased in the early pregnancy, 1 case in the middle pregnancy, and 2 cases slightly increased in the late pregnancy). Because the serum CA125 concentration of pregnant women is obviously affected by pregnancy, especially in the early stages of pregnancy, CA125 ≥60 is taken as the diagnostic threshold. If CA125 ≥60 was taken as the critical value for diagnosis, there were 3 cases (27.3%) of elevation, 1 case of early pregnancy borderline tumor recurrence, 1 case of early pregnancy ectopic pregnancy with borderline tumor, and 1 case of late pregnancy cyst rupture. HE4, CA-199, and CEA are not affected by pregnancy and can be used for monitoring and follow-up of ovarian tumors during pregnancy, but their screening value for tumors is general. All the 8 patients with HE4 were within the normal range. Among the 9 patients with CA-199, 3 were elevated, 1 was significantly elevated >500, 6 were normal, and 2 were slightly elevated among the 10 patients with CEA. The level of serum AFP in 8 patients increased, which was related to the pregnancy status of the patients (**Table 1**). It was generally of diagnostic value for ovarian germ cell tumors but of little significance for the diagnosis of BOT. Due to the lack of clinical data in this group, the diagnostic significance of tumor markers for pregnancy with ovarian borderline tumors still needs to be verified by large sample data.

**Table 1:** Diagnosis of tumor markers.

Gestation	NO	Stage	Gestational weeks	CA125	HE4	CA199	CEA	AFP
First trimester	1	IA	8 w + 4	18.68	36.62	NA	1.23	NA
	2	IB	13 w	151.2	53.18	537.4	7.82	9.89
	3	IA	8 w	50	40.09	57.74	2.27	2.9
	4	IA	7 w + 2	204.7	49.72	NA	0.772	NA
Second trimester	1	IB	15 w + 4	30.2	NA	7.21	0.959	22.76
	2	IB	20 w	90.07	78.65	9.3	0.474	60.16
Third 0trimester	1	IA	36 w + 2	46.13	NA	14.98	0.842	73
	2	IA	35 w + 6	24.31	NA	3.5	0.665	237.1
	3	IA	39 w	NA	NA	NA	NA	NA
	4	IA	39 w + 2	72.65	54.18	14.31	NA	NA
	5	IA	38 w + 3	NA	NA	NA	NA	NA
	6	IB	37 w + 3	NA	NA	NA	NA	NA
	7	IA	39 w + 2	NA	NA	NA	NA	NA
	8	IA	34 w + 3	NA	NA	NA	NA	NA
	9	IC3	39 w + 2	NA	NA	NA	NA	NA
	10	IC2	32 w + 6	32.3	82.05	21.66	5.3	194.9
	11	IC1	28 + w2	25.69	62.12	52.49	0.82	198.4

CA125 (0–35U/mL), HE4 (<140 pmol/L), CA199 (0–37 U/mL), CEA (0–5 ng/mL), AFP (0–9ng/mL) NA, Not Available.

#### 3.2.3 Operation Method

As shown in **Table 2**, of the 17 patients, 4 patients underwent surgery in early pregnancy, of which 2 underwent conservative surgery and induced abortion at the same time; nne patient underwent unilateral adnexal and contralateral cyst enucleation, and delivered a healthy live baby from pregnancy to full-term; one patient with ectopic pregnancy underwent enucleation of the ovarian cyst and right salpingectomy. Two patients who underwent surgery in the second trimester of pregnancy were conservative, and their newborns were healthy from normal pregnancy to delivery. Eleven patients underwent surgery in the third trimester of pregnancy. One patient underwent a right appendectomy due to cyst enlargement at 28 weeks of pregnancy. One healthy live baby was delivered by cesarean section after 38 weeks of pregnancy; one patient underwent cesarean section and right appendectomy at 32 weeks of pregnancy due to cyst rupture; one patient underwent cesarean section, left adnexectomy, and right ovarian dissection due to cyst enlargement; the other 8 patients were accidentally found during cesarean section due to obstetric indications. The newborns survived healthily in the third trimester of pregnancy.

**Table 2:** Operation method and operation time.

Operation mode	First trimester	Second trimester	Third-trimester (No cesarean section)	Third-trimester (simultaneous cesarean section)
Excision of ovarian cyst		1		7
Unilateral appendectomy			1	2
Unilateral appendage or ovariectomy + contralateral cyst removal	1	1		
Unilateral adnexectomy+ contralateral ovarian dissection				1
Unilateral appendectomy + induced abortion	1			
Ovarian cyst excision + induced abortion	1			
Excision of ovarian cyst+ Right salpingectomy	1			

As for the operation method, only 3 patients underwent laparoscopic surgery, 2 patients underwent induced abortion, 1 patient underwent ectopic pregnancy surgery, and the rest underwent open surgery. The guidelines for the diagnosis and treatment of ovarian tumors during pregnancy suggest that both laparotomy and laparoscopy can be performed during pregnancy, and the maternal and fetal outcomes are satisfactory. However, when the mass is large, open surgery is generally recommended, and a large enough longitudinal incision should be selected to fully expose the surgical field of vision to reduce the interference to the uterus.

#### 3.2.4 Pathological Diagnosis

As shown in **Table 3**, of the 17 patients, 3 cases had no intraoperative freezing pathology. Intraoperative freezing suggested borderline in 10 cases, active growth of cystadenoma in 2 cases, and benign in 2. Six cases were completely consistent with postoperative paraffin pathology, 2 cases showed active growth or benign cystadenoma by intraoperative freezing, and postoperative paraffin showed a focal borderline. In other cases, the paraffin pathology was upgraded compared with the intraoperative frozen pathology, and 3 cases showed malignant transformation.

**Table 3:** Intraoperative frozen pathology and postoperative paraffin pathology.

Operation time	No.	Stage	Gestational weeks	Clinical symptoms	Intraoperative frozen pathology	Postoperative paraffin pathology
First trimester	1	IA	8 w + 4	Conscious abdominal distention	Mucinous cystadenoma with active cell growth.	(left) ovarian mucinous cystadenoma with active cell growth and focal borderline (<10%)
	2	IB	13 w	Pelvic mass found	Cyst of left ovary, to be paraffin removed, borderline, mucinous cystadenoma of right ovary, to be paraffin removed	(right) ovarian borderline serous mucinous tumor (left) ovarian serous mucinous tumor, focal borderline
	3	IA	8 w	Pelvic mass found	Papillary cystadenoma, focal junction, to be paraffin	(left) ovarian borderline papillary mucinous cystadenoma with endometriosis
	4	IA	7 w + 2	Menopause, vaginal bleeding	Focal junction of left ovarian cyst	Serous papillary cystadenoma of left ovary, focal junction, < 10% Right fallopian tube pregnancy
Second trimester	1	IB	15 w + 4	Pelvic mass found	Serous papillary cystadenoma, considering focal borderline (area < 10%)	(left) ovarian serous papillary cystadenoma, focal borderline (area < 10%)
	2	IB	20 w	Enlargement of pelvic mass	Borderline cystadenoma	(Right adnexa, left ovary) Borderline serous-mucinous cystadenoma Pelvic lesions: tumor implantation
Third trimester	1	IA	36 w + 2	Elevated blood pressure with ascites	Borderline papillary cystadenoma	(left) ovarian serous borderline papillary cystadenoma
	2	IA	35 w + 6	Huge ovarian cyst found	Mucinous cystadenoma with active cell growth	(left) ovarian mucinous cystadenoma, focal borderline
	3	IA	39 w	Premonitory labor	Consider the left ovarian cystadenoma, where the focal cell proliferation is active, except for paraffin, and the focal junction.	(left) ovarian borderline endometrioid tumor, considering the partial malignant transformation (right) chocolate cyst of ovary
	4	IA	39 w + 2	Elective cesarean section	Borderline papillary cystadenoma	(right) borderline serous papillary cystadenoma of ovary
	5	IA	38 w + 3	Elective cesarean section	Partial borderline mucinous tumor	(right) ovarian mucinous cystadenoma, focal borderline
	6	IB	37 w + 3	oligohydramnios	Left side: cystadenoma, focal epithelial hyperplasia active, except for paraffin, focal junction; Right: considered benign, focal epithelial hyperplasia is active	(Left) Ovarian borderline serous-mucinous papillary cystadenoma (Right) Ovarian serosa-mucinous cystadenoma with focal borderline
	7	IA	39 w + 2	Elective cesarean section	NA	(Left) Ovarian borderline serous mucinous cystadenoma with chocolate cyst
	8	IA	34 w + 3	Vaginal discharge	benign	(Left) Ovarian mucinous cystadenoma, focal borderline (consider <10%)
	9	IC3	39 w + 2	Elective cesarean section	NA	(right) ovarian endometriosis with adenomatous lesion of endometrium at the inner boundary of capsule wall, excluding canceration
	10	IC2	32 w + 6	Rupture of cyst abdominal pain	benign	(Right) Ovarian mucinous cystadenoma, partially borderline
	11	IC1	28 + w2	The cyst is obviously enlarged	NA	(right) ovarian borderline endometrioid tumor, with a tendency of local malignant transformation

NA, Not Available.

The pathological types were mainly divided into the following four types: 4 cases of serous, 5 cases of mucinous, 5 cases of serous mucinous, and 3 cases of endometrioid. Among them, there were 7 cases of focal borderline, 1 case of bilateral borderline and one side focal, and 3 malignant cases or with malignant trend. The pathological types were endometrioid tumors.

Paraffin pathology showed malignant changes in 3 cases after the operation. One patient underwent a right appendectomy at 28 weeks of pregnancy and was pregnant at 38 weeks after the operation. The omentum and appendix were removed at the same time during the cesarean section. In ascites examination, no abnormality was found. Another patient underwent a comprehensive staging operation after pathological consultation in the hospital. The postoperative diagnosis was ovarian malignant tumor in stage IC3 (right ovarian borderline tumor, local malignant transformation into highly differentiated endometrioid carcinoma), and chemotherapy (paclitaxel 240 mg + carboplatin 500 mg) for 3 times without recurrence. One patient underwent comprehensive exploration and staging of ovarian cancer with fertility preservation after cesarean section. The postoperative diagnosis was endometrioid carcinoma of the left ovary, stage IA, with regular postoperative reexamination and no recurrence.

### 3.3 Maternal and Infant Prognosis

As shown in **Table 4**, of the 17 patients, 4 underwent early pregnancy surgery, of which 2 underwent conservative surgery and induced abortion at the same time; one case with tubal pregnancy underwent enucleation of the ovarian cyst and right salpingectomy. After the conservative operation, no staged operation was performed. There was no recurrence after regular review. One case underwent unilateral appendage + contralateral cyst enucleation. One healthy live baby was delivered after normal pregnancy to a full-term cesarean section. The patient did not undergo staged surgery, did not relapse, and the newborn was healthy. Two cases underwent the operation in the middle of pregnancy. A cesarean section was performed from a conservative operation to a full-term pregnancy. There was no staged operation, no recurrence, and the newborn was healthy. Of the 11 cases that underwent surgery in the third trimester of pregnancy, 3 cases showed malignant transformation. One patient underwent comprehensive exploration and staged surgery for ovarian cancer with fertility preservation after cesarean section. The postoperative diagnosis was endometrioid carcinoma of the left ovary, Phase a. Another case underwent a comprehensive staging operation after pathological consultation at an external hospital. The postoperative diagnosis was ovarian malignant tumor in stage IC3 (right ovarian borderline tumor, local malignant transformation into highly differentiated endometrioid carcinoma), and chemotherapy with paclitaxel 240 mg + carboplatin 500 mg for 3 times. One case underwent a right appendectomy at 28 weeks of gestation and was pregnant for 38 weeks. During the cesarean section, the omentum and appendix were removed at the same time. No abnormality was found on the ascites examination. The above 3 cases were rechecked regularly after the operation, and there was no recurrence, and the newborn was healthy.

**Table 4:** Maternal and infant prognosis.

Operation time	No.	Stage	Gestational weeks	Re staged surgery(yes/no)		Maternal prognosis		Neonatal prognosis
					Simultaneous cesarean section/induced abortion/Ectopic pregnancy surgery	Recurrence (yes/no)	Full term pregnancy (yes/no)	Health (yes/no)
First trimester	1	IA	8 w + 4	no	induced abortion	no	–	–
	2	IB	13 w	no	–	no	Conservative operation to full-term pregnancy	Yes
	3	IA	8 w	no	induced abortion	no	–	–
	4	IA	7 w + 2	no	Ectopic pregnancy surgery	no	–	–
Second trimester	1	IB	15 w + 4	no	–	no	Conservative operation to full-term pregnancy	yes
	2	IB	20 w	no	–	no	From conservative operation to 33 weeks of pregnancy, premature rupture of membranes	Premature infants, physical health
Third trimester	1	IA	36 w + 2	no	cesarean section	no	yes	yes
	2	IA	35 w + 6	no	cesarean section	no	yes	yes
	3	IA	39 w	Partial malignant transformation and comprehensive staged operation to preserve reproductive function	cesarean section	no	yes	yes
	4	IA	39 w + 2	no	cesarean section	no	yes	yes
	5	IA	38 w + 3	no	cesarean section	no	yes	yes
	6	IB	37 w + 3	no	cesarean section	no	yes	yes
	7	IA	39 w + 2	no	cesarean section	no	yes	yes
	8	IA	34 w + 3	no	cesarean section	no	premature infant	Premature infants, physical health
	9	IC3	39 w + 2	Except for malignant transformation, comprehensive staged operation was performed	cesarean section	no	yes	yes
	10	IC2	32 w + 6	no	cesarean section	no	premature infant	Premature infants, physical health
	11	IC1	28 + w2	Malignant trend, no comprehensive staging operation	–	no	Conservative operation to full-term pregnancy	yes

## 4 Discussion

### 4.1 General Situation Analysis

Borderline ovarian tumors are more common in young women. Patients with borderline ovarian tumors during pregnancy are younger, which is closely related to gestational age. According to the study by Flicek et al., the average diagnostic age of mucinous borderline tumors was 39.6 years and that of serous borderline tumors was 38.06 years. Up to one-third of patients with marginal tumors are under the age of 40 (4). Among the 17 patients, 16 were under 40 years old, with an average age of 31.82 years (25–45 years). All 17 patients had no specific symptoms. Four cases were found in the early stages of pregnancy, 2 cases in the middle stage of pregnancy, and 11 cases in the late stages of pregnancy. Among them, 9 cases had the main complaint of finding a huge ovarian cyst or cyst enlargement or rupture, and the other 8 cases were accidentally found during cesarean section due to obstetric indications (4).

### 4.2 Diagnosis

#### 4.2.1 Imaging Diagnosis

**Figure 2:**
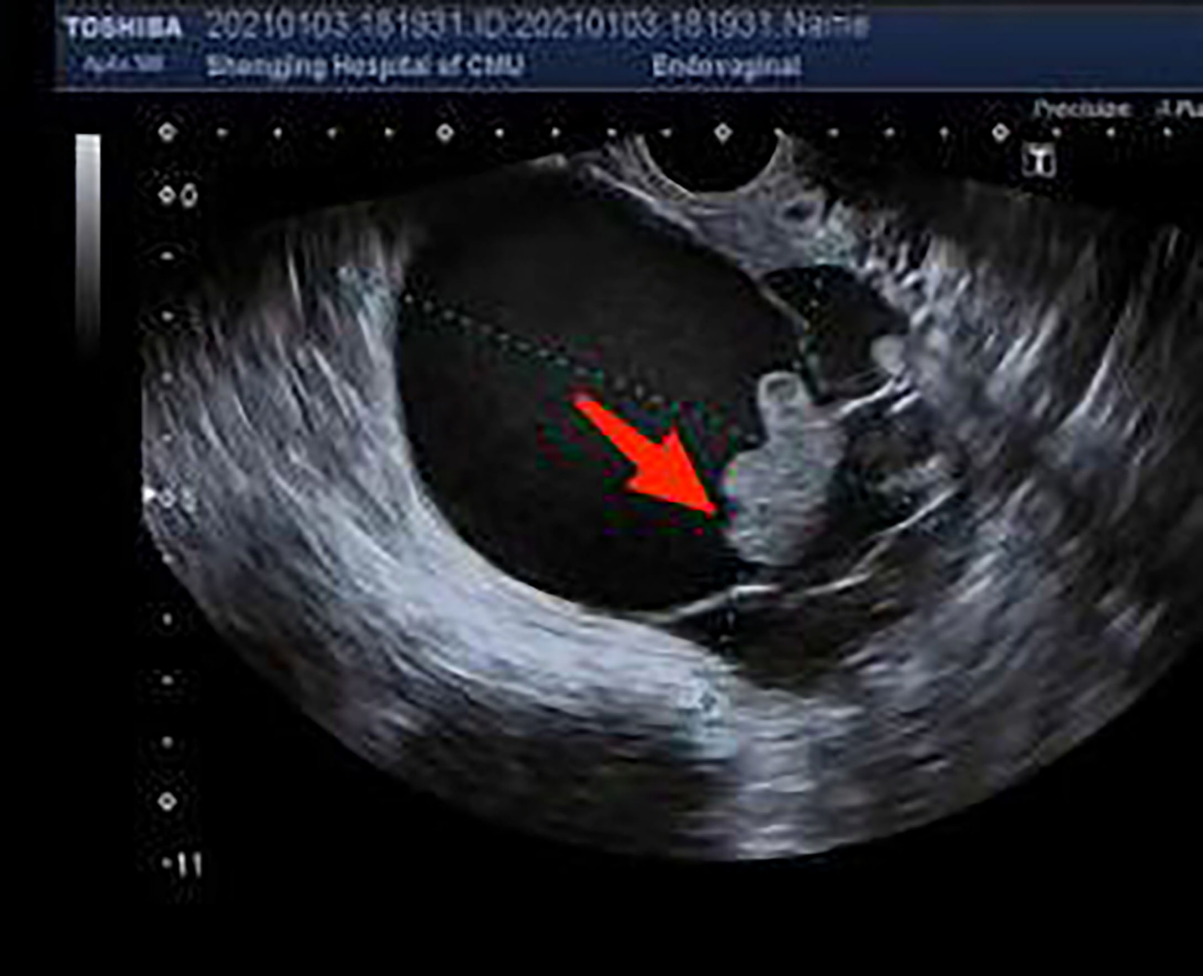
Ultrasound guided microcapsule pattern of borderline ovarian tumors.

Pelvic ultrasound is the most commonly used and repeatedly used imaging examination method during pregnancy. All of the above 17 patients were examined by ultrasonography, but the bilateral adnexal areas were not clearly displayed due to the shielding of the enlarged uterus during pregnancy. Therefore, some ultrasonography did not indicate ovarian tumors. If a cyst in the adnexal area is found during the antenatal examination, the patient should be closely followed up. In the case of cyst enlargement and rupture, it should be diagnosed and treated in time. The common imaging features of borderline ovarian tumors include rare and small internal papillary processes, thin septum, and no clear enhancement. However, the ultrasonic imaging features of bots and benign and malignant ovarian tumors overlap more, and there is a lack of specific ultrasonic manifestations related to bots. It is difficult to distinguish bots from benign and malignant ovarian tumors. Both studies by Zheng and Timor-Tritsch identified microcystic and opacity on ultrasound as independent predictors of borderline ovarian tumors. This “microcapsule pattern” is an ultrasonic marker composed of papillary processes, solid components, and/or septum, which can be observed in most cases of serous and mucinous ovarian borderline tumors (**Figure 2**). According to the comparison of ultrasound images and histopathology between benign entities and malignant tumors, the appearance of microcapsules is unique to ovarian borderline tumors. Therefore, the use of this new marker is helpful in correctly identifying BOT (5, 6).

Among the 17 patients, 4 cases were diagnosed in the early stages of pregnancy, 2 cases showed cystic, and 2 cases showed cystic and solid. In the second trimester of pregnancy, ultrasonography showed cystic and solid. Among the 11 patients in the third trimester of pregnancy, 4 patients did not show abnormalities by prenatal ultrasound, 3 patients showed cystic mass by ultrasound, and the other 4 patients showed cystic solid by preoperative ultrasound. Among them, 3 cases showed that a few moderate echogenic protrusions were seen in the wall of the cyst, and the postoperative pathological results were all of ovarian borderline papillary serous mucinous cystadenoma. It shows that the microcystic pattern under ultrasound has a certain significance in the diagnosis of borderline papillary serous mucinous cystadenoma of the ovary, but it still needs to be confirmed by large sample studies. MRI is also commonly used to diagnose borderline ovarian tumors because of its high resolution of soft tissue. However, in the above cases, only 2 cases of giant cyst and 1 case of large cystic solid mass improved MRI, and the diagnostic value was not fully reflected. According to the research by Flicek et al. on MRI, borderline ovarian tumors are usually round or oval lesions with a clear boundary, which can be pure cystic lesions or cystic lesions with papillary processes and nodules. Cystic and solid lesions are not common (4, 7). Ovarian malignant tumors are mostly unclear boundaries, irregular shapes, and complex cystic and solid lesions. CT can evaluate the solid enhancement components in the tumor and detect local and distant metastasis. However, due to the particularity of pregnancy, only 1 of the 17 patients underwent total abdominal CT examination, and there was no positive prompt on CT examination. Therefore, the significance of CT examination in the diagnosis of pregnancy complicated with an ovarian borderline tumor is very limited.

#### 4.2.2 Diagnosis of Tumor Markers

Serum tumor markers mainly include CA125, HE4, CEA, AFP, and CA199. The possibility of bots and ovarian malignancy can be judged according to the increase in CA125. If CA125 increases significantly, it is deemed ovarian malignancy. HE4, CEA, AFP, and CA199 can be used as reference indices to distinguish bots from ovarian malignancies. However, because of the presence of fetal antigens during pregnancy, related tumor markers such as AFP and CA-125 will increase physiologically and fluctuate with gestational weeks, so the diagnosis of pregnancy complicated by borderline tumors is more difficult. The serum CA125 of pregnant women was significantly affected by pregnancy, especially in the early stage of pregnancy. CA199 and CEA are less affected by pregnancy and can be used for diagnosis, monitoring, and follow-up of pregnancy. Serum AFP detection during pregnancy will increase, which is related to the pregnancy status of patients. It is generally of diagnostic value for ovarian germ cell tumors but of little significance for the diagnosis of BOT. Therefore, tumor markers are not specific detection indicators of bots. It is necessary to combine tumor markers with ultrasound and/or MRI to improve the accuracy of BOT identification. It is more valuable for continuous follow-up of pregnancy and tumor markers. Due to the particularity of pregnancy and the limitations and incompleteness of preoperative examination, the diagnosis of pregnancy complicated with an ovarian borderline tumor is more difficult. Expert consensus also does not recommend the use of any tumor markers to diagnose BOT during pregnancy (3).

#### 4.2.3 Pathological Diagnosis

The accuracy of preoperative diagnosis of borderline ovarian tumors is low, and the difficulty of diagnosis is further increased because of the influence of pregnancy. A large number of patients are diagnosed with intraoperative frozen pathology. The diagnostic accuracy of intraoperative frozen pathology in benign and malignant ovarian tumors is very high, but due to the limitations of frozen materials, the diagnostic accuracy of borderline ovarian tumors is relatively low (6). According to the study by Basara et al., the total coincidence rate between intraoperative freezing results and postoperative pathological routine results in bots is 79%, of which sbot is 92% and mBot is 62% (8). Among the 17 cases above, 3 cases had no intraoperative freezing pathology, of which 10 cases showed borderline freezing, 2 cases showed active growth of cystadenoma, and 2 cases showed benign. Six cases were completely consistent with postoperative paraffin pathology, 2 cases showed active growth and benign cystadenoma, and postoperative paraffin showed a focal borderline. Compared with the total coincidence rate of 79% shown in relevant studies, this may be related to the small number of cases. Because the intraoperative findings cannot be distinguished from benign tumors, some cases have no intraoperative frozen pathology, and the postoperative pathology suggests a malignant transformation, so the importance of the frozen sections should be paid attention to.

The common histopathological types of borderline ovarian tumors are serous, mucinous, and other rare types, such as serous mucinous, endometrioid, clear cells, and Brenner tumors. Studies have proved that the accuracy of frozen pathology in mucinous tumors is lower than that of other histological types, which is related to the large volume and high heterogeneity of mucinous tumors (9). Among the 5 cases of mucinous borderline tumors, 4 cases were sent for intraoperative frozen pathology, of which 3 cases were upgraded by paraffin pathology after the operation; among the 5 cases of serous mucinous borderline tumors, 3 cases were upgraded by paraffin pathology after the operation. Among the pathological diagnoses of the 17 patients, 4 cases were serous, 5 cases were mucinous, 5 cases were serous mucinous, and 3 cases were endometrioid. Three cases of endometrioid tumors showed malignant transformation. Whether the malignant transformation of borderline tumors is related to the pathological type needs further study.

### 4.3 Treatment and Prognosis

Surgical treatment is the first choice for borderline ovarian tumors. Patients with borderline ovarian tumors in pregnancy are younger and have a good prognosis. Therefore, preserving fertility is an important consideration in the choice of treatment scheme (10). However, compared with radical surgery, fertility-preserving surgery has a higher recurrence rate (1), which is comprehensively estimated by a systematic review report to be 13%, while the recurrence rate after radical surgery is 0–5%, but most recurrences show no malignant change (11). Among the 17 patients in this paper, 3 patients with malignancy suggested by postoperative paraffin pathology underwent comprehensive staged surgery again, and the rest underwent fertility-preserving surgery. Through the postoperative outpatient review records and telephone follow-up, the longest follow-up time was 89 months and the shortest follow-up time was 9 months. All 17 patients had a good prognosis, with no recurrence during the follow-up period, and all newborns survived healthily. A French multicenter study by Zillioxd et al. also shows that conservative surgery is safe and effective for patients with pregnancy complicated by borderline ovarian tumors (3). The neonatal Apgar score was related to gestational age and obstetric complications, but not to borderline ovarian tumors. Ovarian borderline tumor disease has slow progress and good prognosis, most of which are long-term recurrences, and some patients recur 20 years later (12). Therefore, patients undergoing conservative surgery still need long-term follow-up. Due to the small number of cases, no effect of appendectomy and cyst removal on the prognosis of patients was found. Fang et al. believe that unilateral adnexectomy is the first choice for patients with unilateral borderline ovarian tumors to obtain ideal oncological results and a satisfactory pregnancy rate; bilateral cyst resection should be the first choice for patients with bilateral borderline ovarian tumors. Further research is needed to study the effects of different types of fertility-preserving surgery on postoperative recurrence rate and prognosis. Except for the 3 patients with malignant tumors indicated by paraffin pathology after the operation, the other patients did not undergo comprehensive staged operations after the operation and did not receive chemotherapy after the operation. There has been no recurrence since follow-up. However, bots have the tendency for long-term recurrence, and more than 10 years of long-term follow-up is still needed after the conservative operation.

## 5 Conclusion

The preoperative diagnosis of borderline ovarian tumors is delayed, and the imaging and tumor markers are not specific. Due to the limitations of pregnancy, the relevant examinations cannot be carried out. However, the adnexal cysts found during a routine prenatal examination should be closely followed up. Once the cysts are found to be enlarged or broken, they should be operated on as soon as possible. Most borderline ovarian tumors are initially diagnosed by intraoperative frozen pathology, but the coincidence rate between intraoperative frozen pathology and postoperative paraffin pathology is not high. According to postoperative paraffin pathology, it may be necessary to further expand the scope of surgery. Therefore, the diagnosis of ovarian borderline tumors during pregnancy is still a great challenge. Pregnancy with an ovarian borderline tumor is mainly treated by surgery. The prognosis is good, but the recurrence cycle is long. Long-term follow-up is still needed after the operation.

## Data Availability Statement

The original contributions presented in the study are included in the article/supplementary material. Further inquiries can be directed to the corresponding author.

## Ethics Statement

The study was approved by the Ethics Committee of Shengjing Hospital, of the Chinese Medical University, on 31 October 2008 (Ethics No. 2018PS175J). The patients/participants provided their written informed consent to participate in this study.

## Author Contributions

MW is the first author and wrote the manuscript. KL edited the manuscript, and approved the final version. TX, YL, CS, and LJ consulted references and collected clinical data. All authors ultimately agreed to submit this article. All authors listed have made a substantial, direct, and intellectual contribution to the work and approved it for publication.

## Funding

The authors thank the support of the “Scientific research funding project of Liaoning Provincial Department of Science and Technology (No. 2020JH2/10300050) for this article.

## Conflict of Interest

The authors declare that the research was conducted in the absence of any commercial or financial relationships that could be construed as a potential conflict of interest.

## Publisher’s Note

All claims expressed in this article are solely those of the authors and do not necessarily represent those of their affiliated organizations, or those of the publisher, the editors and the reviewers. Any product that may be evaluated in this article, or claim that may be made by its manufacturer, is not guaranteed or endorsed by the publisher.
